# Comparison of Automated Extraction Techniques for Volatile Analysis of Whole Milk Powder

**DOI:** 10.3390/foods10092061

**Published:** 2021-09-01

**Authors:** Zeng Cheng, David T. Mannion, Maurice G. O’Sullivan, Song Miao, Joseph P. Kerry, Kieran N. Kilcawley

**Affiliations:** 1 Food Quality and Sensory Science, Teagasc Food Research Centre, Moorepark, P61 C996 Cork, Ireland; Zeng.Cheng@teagasc.ie (Z.C.); david.mannion@teagasc.ie (D.T.M.); 2 Sensory Group, School of Food and Nutritional Sciences, University College Cork, T12 R229 Cork, Ireland; maurice.osullivan@ucc.ie; 3 Department of Food Chemistry and Technology, Teagasc Food Research Centre, Moorepark, P61 C996 Cork, Ireland; song.miao@teagasc.ie; 4 China-Ireland International Cooperation Centre for Food Material Science and Structure Design, Fujian Agriculture and Forestry University, Fuzhou 350002, China; 5 Food Packaging Group, School of Food and Nutritional Sciences, University College Cork, T12 R229 Cork, Ireland; joe.kerry@ucc.ie

**Keywords:** whole milk powder, automated volatile extraction, gas chromatography mass spectrometry, HiSorb, headspace solid phase microextraction, thermal desorption

## Abstract

Volatile profiling of whole milk powder is valuable for obtaining information on product quality, adulteration, legislation, shelf life, and aroma. For routine analysis, automated solventless volatile extraction techniques are favored due their simplicity and versatility, however no single extraction technique can provide a complete volatile profile due to inherent chemical bias. This study was undertaken to compare and contrast the performance of headspace solid phase microextraction, thermal desorption, and HiSorb (a sorptive extraction technique in both headspace and direct immersion modes) for the volatile analysis of whole milk powder by gas chromatography mass spectrometry. Overall, 85 unique volatiles were recovered and identified, with 80 extracted and identified using a non-polar gas chromatography column, compared to 54 extracted, and identified using a polar gas chromatography column. The impact of salting out was minimal in comparison to gas chromatography column polarity and the differences between the extraction techniques. HiSorb extracted the most and greatest abundance of volatiles, but was heavily influenced by the number and volume of lactones extracted in comparison to the other techniques. HiSorb extracted significantly more volatiles by direct immersion than by headspace. The differences in volatile selectivity was evident between the techniques and highlights the importance of using multiple extraction techniques in order to obtain a more complete volatile profile. This study provides valuable information on the volatile composition of whole milk powder and on differences between extraction techniques under different conditions, which can be extrapolated to other food and beverages.

## 1. Introduction

The global production of whole milk powder (WMP) was 10.8 million tons in 2019 and is anticipated to reach 13.2 million tons by 2024 [1]. It remains a considerable export product for Ireland with 57,000 tons exported in 2019 [2]. Dairy powders such as WMP have unique flavor characteristics that are heavily influenced by fat content and fat distribution [3], but are also very susceptible to lipid oxidation [4]. Many studies on the volatile properties of dairy products have only evaluated single extraction techniques. However, as all extraction techniques have inherent bias towards certain volatiles based upon the properties of the volatile organic compounds (VOCs), their affinity to the sample matrix, and the properties and parameters of the extraction technique [5], it is therefore useful to evaluate a wider range of extraction techniques in order to get the best possible representative volatile profile of a sample.

Arguably the most widely used volatile extraction technique to date is solid phase microextraction (SPME), mainly due to its versatility, ease of use (as it is fully automatable), the wide range of coating materials available (single, dual, or multiple phases in different thicknesses), and its general robustness. It can be used as a direct immersion (DI) technique or, most commonly, as a headspace (HS) technique. Headspace solid phase microextraction (HS-SPME) is a static HS technique that has been extensively applied to analyze VOCs in dairy products, with the divinylbenzene/carboxen/polydimethylsiloxane (DVB/CAR/PDMS) fiber finding the greatest use, due to its potential ability to capture a broader range of VOCs owing to the inherent properties of each phase [6,7,8]. However, the relatively limited surface capacity of the fiber can result in competition between analytes for adsorption/absorbtion sites and displacement resulting in increased bias for certain VOCs [9]. Thermal desorption (TD) is a well-established dynamic extraction technique, where an inert carrier gas strips the volatiles from a sample where they are subsequently trapped in a sorbent packed tube with absorbent/adsorbent material [10,11]. The main advantages are the wide range of sorbent phases available and the large capacity of sorbent phase. However, managing moisture can be problematic, and this may be why its use in dairy applications is limited [12,13]. Tenax (TEN) is typically the most widely used sorbent material in TD because of its affinity for VOCs with a very wide range of boiling points between 60 °C and 300 °C [14]. Recently, another passive sorbent extraction technique was developed called HiSorb™ (Markes International Ltd., Llantrisant, UK). It is somewhat similar to stir bar sorptive extraction (SBSE) [15] and can also be performed as a headspace (HS) or as a direct immersion (DI) technique. With HiSorb to date, a single sorbent phase polydimethylsiloxane (PDMS) is coated in a specially designed probe that can be either exposed to a HS above a sample or directly immersed in a liquid sample under controlled conditions. After exposure, the probe is placed in an empty sorbent tube and treated in a similar manner to a TD sorbent tube, where it is desorbed.

In terms of sample preparation, “salting out” is a useful practice to potentially increase the extraction efficiency of certain volatile analytes. Salt, usually sodium chloride (NaCl), is added to the sample, which reduces the solubility of hydrophobic compounds, resulting in decreased water availability and thus, in theory, making polar and low molecular weight VOCs easier to extract [16]. The polarity of the gas chromatography (GC) column is also an important factor in relation to the separation of individual VOCs. The most common types are polar and non-polar phases, both of which offer better separation and resolution for specific chemical classes [17], with non-polar phases having greater stability. Therefore, in order to obtain the best possible volatile profile, it is also useful to assess both polar and non-polar GC columns.

In this study, we compared the ability of four automated volatile extraction techniques (HS-SPME, TD, and HiSorb as HS (HS-HiSorb) and as DI (DI-HiSorb)) for their ability to profile volatile compounds in WMP using gas chromatography mass spectrometry (GC-MS). Each extraction technique was assessed with or without salting out and using both a polar and non-polar GC column.

## 2. Materials and Methods

### 2.1. Preparation of Whole Milk Powder

Raw milk was produced from 54 Friesian cows at the Teagasc Moorepark dairy farm, Fermoy Co., Cork, Ireland. The milk was pre-heated to 50 °C in an APV plate heat exchanger (SPX Flow Technology, Crawley, West Sussex, UK), separated by a centrifugal disk separator, and pasteurized at 72 °C for 15 s. The pasteurized milk was subsequently preheated to 78 °C and evaporated in a Niro three-effect falling film evaporator (GEA Niro A/S, Soeborg, Denmark) at sequential effect temperatures of 73 °C, 64 °C, and 55 °C. Concentrate feed was introduced to a Niro Tall-Form Anhydro three-stage spray dryer (GEA Niro A/S, Soeborg, Denmark) (air inlet temperature = 180 °C and air outlet temperature = 85 °C) at approximately 43% total solids (TS) with a centrifugal atomizer (GEA Niro A/S, Soeborg, Denmark) at Moorepark Technology Ltd. (Fermoy Co., Cork, Ireland). Primary and secondary fluidized beds were maintained at 74 °C and 24 °C, respectively. Fines were returned to the cyclone to the top of the spray dryer. WMP samples were stored at room temperature in sealed 900 g aluminum vacuum cans until analysis.

WMP samples were dissolved at 10% solids (*w*/*v*) using ultra-pure deionized water and stored at 4 °C overnight to ensure solubility, without overhead lights to prevent light-induced off-flavor formation. Each extraction technique was assessed with or without salting out. NaCl (0.75 g) (Merck, Co., Wicklow, Ireland) was added to 5 mL of the 10% *w*/*v* WMP sample, equivalent to 15% NaCl *w*/*v*. This was mixed until soluble (~30 min).

### 2.2. Internal and External Standard Preparation

To monitor the performance of the GC-MS operating conditions, an external standard (ES) solution was added at the start and end of each GC-MS sample run. The ES was comprised of 1-butanol, dimethyl disulfide, butyl acetate, cyclohexanone, and benzaldehyde (Merck, Ireland) at 10 mg L^−1^ with 2-phenyl-D5-ethanol (Merck, Arklow, Co., Wicklow, Ireland) added at 5 mg L^−1^ in ultra-pure water. For the HS-SPME technique, 10 µL of ES was added to the sample in a 20 mL amber La-Pha-Pack HS vial with magnetic screw caps and a silicone/polytetraflurorethylene septa (Apex Scientific Ltd., Maynooth, Ireland); see details in Section 2.3.1. The ES (10 µL) was also added to the TD tube containing the sample extract for both TD and HiSorb (HS-HiSorb and DI-HiSorb), the details of which are described in Section 2.3.2 and Section 2.3.3. To monitor the performance of each extraction procedure, an internal standard (IS) of 2-phenyl-D5-ethanol and 4-methyl-2-pentanol (Merck, Arklow, Co., Wicklow, Ireland) at 20 mg L^−1^ in ultra-pure water, was added (50 µL) to each WMP sample prior to extraction.

### 2.3. Extraction Procedures

The following codes were used to describe each extraction technique with and without salting out for both polar and non-polar GC columns (Table 1).

An extraction temperature of 40 °C was used for each technique based on previous experience and to ensure sufficient VOC extraction without creating additional VOC due to Maillard reactions or caramelization during the extraction process. The extraction times varied between techniques based on specific aspects of each technique and on previous experience. An equilibration step was necessary for the HS-SPME to maximize the VOC concentration in the HS prior to extraction.

#### 2.3.1. Head-Space Solid Phase Microextraction (HS-SPME)

The WMP solutions (5 mL) were added to a 20 mL amber La-Pha-Pack vial (as described in Section 2.2) and equilibrated to 40 °C for 10 min, with pulsed agitation of 5 s at 500 rpm using an Agilent GC 80 Autosampler (Agilent Technologies Ireland Ltd., Cork, Ireland). Each sample was pre-incubated at 40 °C with pulsed agitation for 10 min. A single SPME 50/30 μm (DVB/CAR/PDMS) fiber (Agilent Technologies Ireland Ltd., Cork, Ireland) was exposed to the headspace above the samples in the vial for 20 min at a depth of 1 cm at 40 °C. Following extraction, the SPME fiber was retracted and injected into the gas chromatograph inlet and desorbed for 3 min at 250 °C in splitless mode. The fiber was cleaned in a bakeout conditioning station (Agilent Technologies Ireland Ltd., Cork, Ireland), between each sample injection, at 270 °C with a nitrogen flow of 6 mL min^−1^, and blanks were conducted after every triplicate sample to ensure no carryover occurred. A Merlin microseal (Agilent Technologies Ireland Ltd., Cork, Ireland) was used to minimize fiber wear. Each sample was analyzed in triplicate.

#### 2.3.2. Thermal Desorption Extraction

A micro-chamber/thermal extractor (Markes International Ltd., Llantrisant, UK) was used for dynamic headspace extraction using industry standard TD tubes packed with Tenax/Carbograph (TEN/CAR) (Markes International Ltd., Llantrisant, UK). The analysis was undertaken in triplicate and the TEN/CAR tubes were preconditioned at 280 °C for 1 hr prior to sampling using a TC-20 (Markers International Ltd., Llantrisant, UK). A Unity 2 thermal desorption unit (Markes International Ltd., Llantrisant, UK) was used to concentrate the volatiles and remove excess moisture. A heated transfer line was used to automatically transfer the volatiles from the Unity 2 to the GC. The WMP solution (5 mL), containing the IS, was added to an inert stainless steel microchamber pot and extracted in the micro-chamber at 40 °C at 50 mL min^−1^ in nitrogen for 20 min. Each sorbent tube was desorbed in the Unity 2 thermal desorption unit with a materials emission focusing trap (Markes International Ltd., Llantrisant, UK). The sample tubes were initially pre-purged for 2 min with a 1:20 split, followed by a two-stage desorption. In the first stage, the tubes were ramped to 110 °C with a 1:10 split for 10 min, then heated to 280 °C for 10 min without a split. The cold trap was set at 30 °C, with a trap flow of 50 mL min^−1^. After tube desorption, a pre-trap fire purge was performed for 2 min, before heating the trap to 300 °C at 100 °C s^−1^ for 5 min without a split. The transfer line was held at 160 °C. Each sample was analyzed in triplicate.

#### 2.3.3. Headspace and Direct Immersion Hi-Sorb Extraction

The WMP samples (5 mL) were pipetted into a 20 mL amber La-Pha-Pack vial (Apex Scientific Ltd, Maynooth, Co., Kildare, Ireland) with a HiSorb-P1 inert PDMS probe assembly (Markes International Ltd., Llantrisant, UK) for both HS-HiSorb and for DI-HiSorb. For DI-HiSorb, the HiSorb probe was directly immersed in the liquid WMP sample and sealed. For HS-HiSorb, the probe was placed at a fixed depth of 1 cm above the sample in the vial (care was taken to ensure that the probe remained dry) and sealed. The vials were added to the HiSorb Agitator (Markes International Ltd., Llantrisant, UK) and agitated at 250 rpm for 120 min at 40 °C for the DI-HiSorb. The vials were added to the HiSorb agitator at 250 rpm for 180 min at 40 °C for the HS-HiSorb. The HiSorb probes were rinsed with deionized water and gently dried with a lint-free tissue prior to insertion into a clean, empty TD tube (Markes International Ltd., Llantrisant, UK), which were end capped using brass long-term storage caps (Markes International Ltd., Llantrisant, UK). The TD tubes were then evaluated in an identical manner to that described for the TD extraction. Each HiSorb probe was preconditioned at 280 °C for 1 h between samples using a U-CTE micro-chamber/thermal extractor (Markes International Ltd., Llantrisant, UK).

### 2.4. GC-MS Analysis

The GC-MS system was an Agilent 7890A GC and Agilent 5977B MSD (Agilent Technologies Ireland Ltd., Cork, Ireland). The analysis was undertaken using both a non-polar GC column DB5-MS (60 m × 0.25 mm × 0.25 µm) and a polar GC column HP-Innowax (60 m × 0.25 mm × 0.5 µm) (Agilent Technologies Ireland Ltd., Cork, Ireland). The GC conditions for the non-polar DB5-MS column were as follows: the injector temperature was set at 250 °C, while the column was initially at 35 °C, then increased to 230 °C at 6.5 °C min^−1^, 320 °C at 15 °C min^−1^, before being held for 5 min, yielding a total run time of 41 min. The carrier gas helium was held at a constant pressure of 23 psi. The GC conditions for the polar HP-Innowax column were as follows: the injector temperature was set at 250 °C, while the column was initially at 40 °C for 5 min, then increased to 230 °C at 5 °C min^−1^, before being held for 10 min, yielding a total run time of 59 min. The carrier gas helium was held at a constant pressure of 23 psi.

The ion source temperature was 220 °C and the interface temperature was set at 260 °C. The mass spectrometer was in electronic ionization (70 v) mode with the mass range scanned between 35 and 250 amu. The analysis was undertaken using MassHunter Qualitative Analysis software (Agilent Technologies, Palo Alto, CA, USA) with target and qualifier ions and linear retention indices for each compound compared to an in-house library based on mass spectra obtained from NIST 2014 mass spectral library MS searching (v.2.3, Gaithersburg, MD, USA) and an in-house library created using authentic compounds with target and qualifier ions and linear retention indices for each compound using the Kovats index. Spectral deconvolution was also performed to confirm identification of compounds using the automated mass spectral deconvolution and identification system (AMDIS). Batch processing of the samples was carried out using metaMS [18], an open-source pipeline for GC-MS based untargeted metabolomics. The results for each identified volatile compound were normalized based on the recovery of the 4-methyl-2-pentanol IS for each sample and expressed as a percent of the total volatiles recovered for each sample. Results in all cases were the averages of triplicate analysis.

### 2.5. Data Analysis

Each extraction technique, with or without salting out, were compared using non-polar and polar GC columns in relation to their ability to extract VOCs in these WMP samples. The results were expressed after normalization in relation to the IS. The sensitivity, selectivity, and reproducibility were compared in terms of: (i) the number of VOCs extracted by each technique, (ii) the percentage of each chemical class extracted by each technique, (iii) the specific identity of each VOC extracted by each technique, (iv) the total abundance of VOCs extracted by each technique (the overall abundance was calculated as the sum of the average abundance of every VOC peak area extracted by that technique, and expressed as a percentage. The extraction technique with the highest total abundance equated to 100% and the others were expressed as a percentage thereof), and (v) the average percentage relative standard deviation of each technique (taken from the relative standard deviation achieved for every VOC recovered in triplicate for each technique) as outlined in [19]. Principal component analysis (PCA) biplots of the volatile data were carried out to aid the visual association of volatile compounds using the “factoextra” and “FactoMineR” packages within R (v 3.4.1, R Foundation for Statistical Computing, Vienna, Austria). To visualize the selectivity of each technique in relation to the number of VOCs recovered, with or without salting out using the non-polar and polar GC columns, Venn diagrams were created with the 4 oval flower model using the Excel template (Microsoft Office, Redmond, WA, USA). Histograms outlining the percent of chemical classes of each extraction technique with or without salting out using the non-polar and polar GC columns were also created using Excel (Microsoft Office, Redmond, WA, USA).

## 3. Results and Discussion

### 3.1. Comparison of Volatile Compounds Extracted from Whole Milk Powder by Each Technique

A summary of all the VOCs identified by each technique, with and without salting out, in terms of percent of abundance, including standard deviations for each VOC, are provided in Table 2a (results using a non-polar GC column) and Table 2b (results using a polar GC column). In total, the number of individual VOCs identified in these samples across all four extraction techniques, with and without salting out and with both GC column polarities, was 85 (Table 3). This is considerably more VOCs than previously found in WMP, which, albeit, only used a single extraction technique [20,21]. Twenty-five VOCs were identified using salting out with SBSE (PDMS) using a non-polar GC column [21] and ten VOCs by HS-SPME (DVB/CAR/PDMS) with salting out using a non-polar GC column [20]. The 85 VOCs identified in this study consisted of 20 ketones, 18 aldehydes, 11 lactones, 11 alcohols, 7 esters, 6 benzene/phenols, 5 furans, 4 terpenes, 2 sulphur compounds, and 1 acid. Most VOCs were identified using the non-polar GC column (80) as opposed to the polar GC column (54) across all extraction techniques, independent of salting out (Table 3). A previous study comparing four volatile extraction techniques on natto (a fermented food) also found considerably more volatiles using a non-polar GC column than a polar GC column; 70 compared to 47 VOCs, with 40 VOCs recovered by both column polarities [22]. In this study, 30 (ethanol, 1-butanol, 1-octanol, (Z)-4-heptenal, (E)-2-octenal, (E)-2-nonenal, (E)-2-decenal and undecanal, benzeneacetaldehyde, benzyl alcohol, diacetyl, 2-hexanone, 2-octanone, 3-octanone, 2-tridecanone, 2-pentadecanone, 2-heptadecanone, γ-crotonolactone, δ-caprolactone, δ-nonalactone, γ-dodecalactone, δ-undecalactone, δ-tridecalactone, z-dairylactone, longifolene, methyl hexanoate, methyl pyruvate, 2-methyl furan, 2-pentyl furan, and acetic acid) VOCs were extracted, independent of salting out, using the non-polar GC column in comparison to the polar GC column across all four extraction techniques (Table 2a,b). In contrast, only six (1–3-pentanol, 1-nonanol, 2,3-pentanedione, δ-caprolactone, butyl acetate, and 2-ethyl furan) VOCs were extracted across all four extraction techniques, independent of salting out, on the polar GC column, but not on the non-polar GC column (Table 2a,b). Therefore, the VOCs were present in the extract(s) in each case, but did not interact with the particular GC column phase in order to be identified. This further highlights the significance of GC column polarity in volatile extraction/identification by GC-MS.

More VOCs were extracted and identified across all four techniques with salting out (75) and without salting out (72) with the non-polar GC column, than with salting out (48) and without salting (45) on the polar GC column (Table 4). Therefore, the impact of salting out was much less than the impact of column polarity in relation to the number of VOCs extracted. In general, salting out modifies the ionic strength of the sample solution with the aim of improving the extraction of polar VOCs, but may adversely impact the extraction of non-polar VOCs [23]. However, in practice the impact of salting out in relation to polar and non-polar VOCs is often unclear as many additional factors relating to the composition of the sample and the parameters of the specific extraction technique may also influence the extraction [23].

### 3.2. The Percentage of Each Chemical Class Extracted from Whole Milk Powder by Each Technique

**Figure 1:**
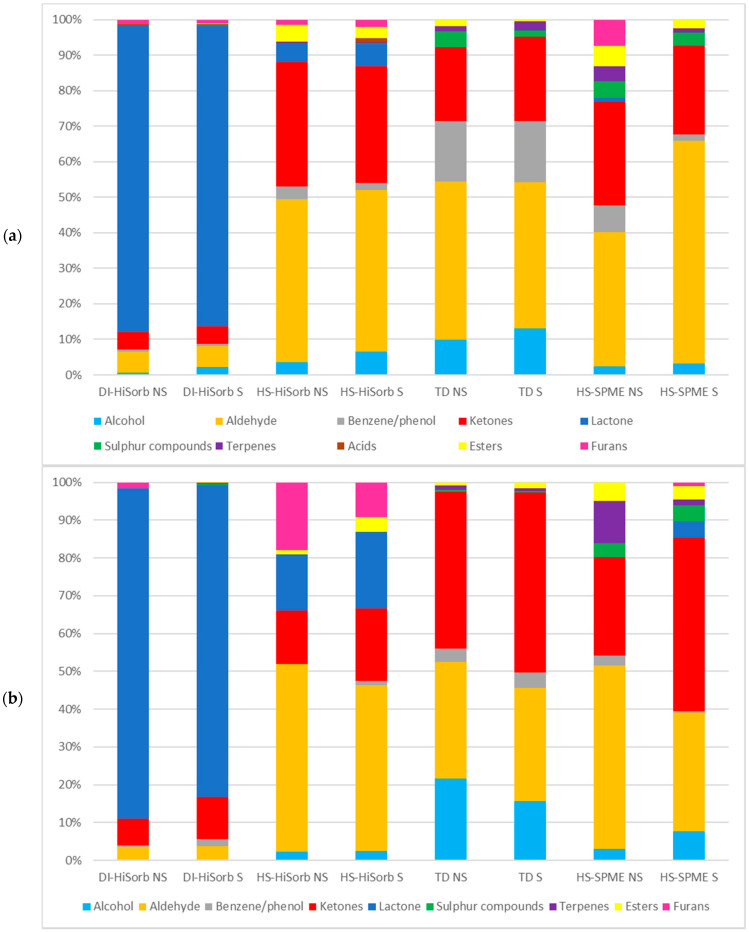
(**a**) The percentage of each chemical class extracted by the four extraction techniques using the non-polar GC column with and without salting out, and (**b**) the percentage of each chemical class extracted by the four extraction techniques using the polar GC column with and without salting out. Direct Immersion HiSorb without salting out (DI-HiSorb NS), Direct Immersion HiSorb with salting out (DI-HiSorb S), Headspace HiSorb without salting out (HS-HiSorb NS), Headspace HiSorb with salting out (HS-HiSorb S), Thermal Desorption without salting out (TD-NS), Thermal Desorption with salting out (TD S), Headspace Solid Phase Microextraction without salting out (HS-SPME NS), Headspace Solid Phase Microextraction with salting out (HS-SPME S).

Figure 1a is histogram highlighting the breakdown of the percentage of each chemical class extracted by each of the four techniques using the non-polar GC column with and without salting out. Figure 1b is the corresponding figure for the polar GC column. It is immediately apparent that significant differences existed in relation to the type and percentage of each chemical class extracted by each technique, influenced by GC column polarity and, to a lesser extent, salting out. All DI-HiSorb techniques (DI-HiSorb S and DI-HiSorb NS), independent of GC column polarity, were characterized by the large volume of lactones extracted (>82%), which differs considerably to all of the other extraction techniques. A similar result was found for SBSE, which is a comparable technique to DI-HiSorb that also used PDMS as the sorbent phase [19]. The only other significant number of chemical classes extracted by DI-HiSorb NS were aldehydes, ketones, and furans, but DI-HiSorb NS did not extract any alcohols, sulphur compounds, acids, terpenes, or esters with the non-polar GC column (but did extract low levels, from 0.5–0.63%, with the polar GC column). The DI-HiSorb S slightly modified the percentage recovery of some chemical classes in comparison to the DI-HiSorb NS. The percentages of chemical classes extracted by DI-HiSorb (DI-HiSorb S and DI-HiSorb NS) using the non-polar GC column were similar independent of salting out (although slightly more alcohols were extracted with salting out). The percentage of each chemical class extracted by HS-HiSorb (HS-HiSorb S and HS-HiSorb NS) for each GC column polarity differed considerably to that attained by DI-HiSorb. This same trend was also apparent when comparing HSSE (similar to HS-HiSorb) and SBSE (similar to DI-HiSorb) [19]. HS-HiSorb S and HS-HiSorb NS attained a much lower percentage of lactones on the polar GC column (~14–20%) and with the non-polar GC column (~5–6%) than DI-HiSorb S and DI-HiSorb NS. HS-HiSorb S and HS-HiSorb NS had a much higher percentage recovery of aldehydes (~42–48%) than DI-HiSorb S and DI-HiSorb NS across both GC column polarities. The percentage of ketones extracted by HS-HiSorb S and HS-HiSorb NS varied considerably depending upon GC column polarity with levels at ~32–35% with the non-polar GC column, and ~14–19% with the polar GC column. The greatest difference in the percentage recovery of chemical classes between HS-HiSorb S and HS-HiSorb NS in relation to GC column polarity, apart from lactones, was for the recovery of furans, with only 1–2% recovered using the non-polar GC column, but 9–17% recovered on the polar GC column. The percentage recovery of esters by HS-HiSorb S and HS-HiSorb NS were relatively high in comparison to the other extraction methods at ~3–5% for the non-polar GC column and ~2–4% for the polar GC column. HS-HiSorb NS did not recover any terpenes, sulphur compounds, acids, or benzene/phenol compounds using the polar GC column, nor sulphur compounds or acids using the non-polar GC column. TD S and TD NS were characterized as having a high percentage recovery of aldehydes (~30–45%), ketones (~21–48%), and alcohols (~10–22%) that varied with both GC column polarity and salting out. TD, independent of salting out, recovered the highest percentage of benzene/phenol compounds using the non-polar GC column in comparison to all the other extraction techniques. TD S or TD NS did not extract any lactones, acids, or furans independent of salting out or GC column polarity. The impact of salting out was minimal in relation to TD, however the percentage of alcohols decreased with salting out using the polar GC column, but increased using the non-polar GC column. Overall, the combination of TEN/CAR should enable a wide range of VOCs to be recovered, as TEN is particularly suited to the extraction of non-polar and slightly polar VOCs, apart from very low molecular weight (<C6) VOCs, which CAR can extract [24]. The percentage recovery of many chemical classes differed most in relation to both GC column polarity and salting out for HS-SPME than any of the other extraction techniques. Overall, HS-SPME recovered all chemical classes except for acids, independent of salting out and GC column polarity, or furans and lactones by HS-SPME NS using the polar GC column, or furans by HS-SPME S using the non-polar GC column. Overall HS-SPME was characterized by a high percentage recovery of aldehydes (~31–62%), which was reduced with the inclusion of salting out independent of both GC column polarities. For HS-SPME S, the percentage recovery of ketones (25–46%) was much higher using the polar GC column. HS-SPME NS also recovered many more terpenes independent of GC column polarity. Previous studies noted that the DVB/CAR/PDMS multiphase SPME fibers tend to extract the most volatile low boiling point VOCs more effectively [25], which corresponds with the results of this study.

No acids were recovered by any technique using the polar GC-column, however it worth pointing out that only one acid (acetic acid) was identified in these WMP samples.

### 3.3. The Relationship between the Individual Volatile Compound Chemical Classes Extracted by Each Technique in the Whole Milk Powder

**Figure 2:**
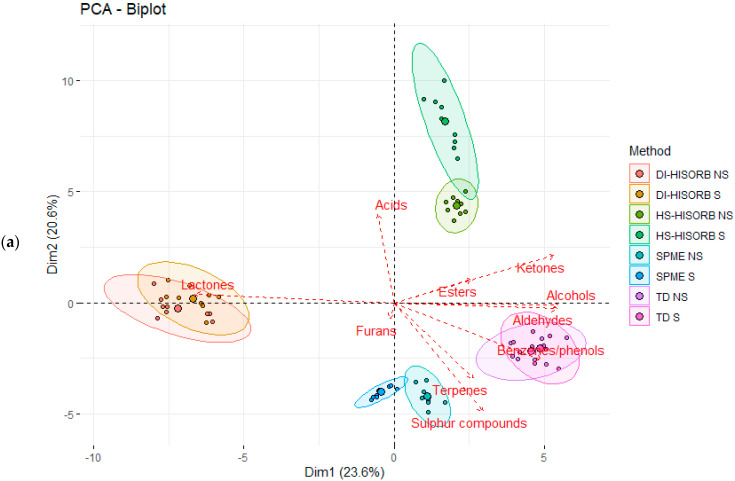

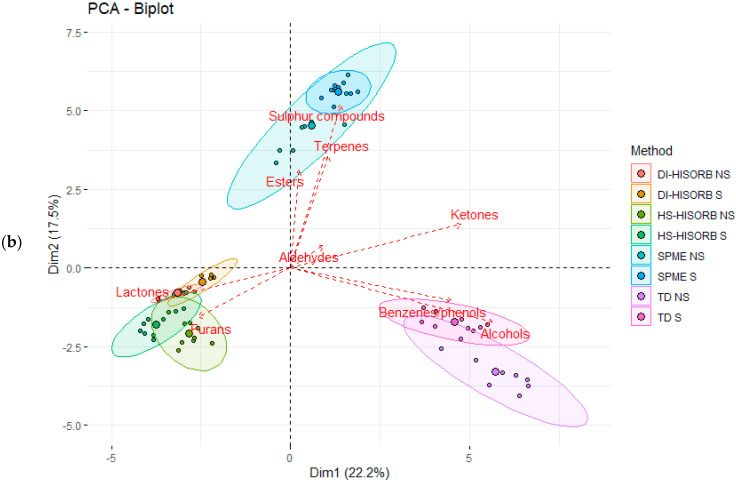
(**a**) Principal component analysis of the volatile organic compounds (as per chemical class) per individual extraction technique, with and without salting out, using the non-polar GC column, and (**b**) principal component analysis of the volatile organic compounds (as per chemical class) per individual extraction technique, with and without salting out, using the polar GC column. Direct Immersion HiSorb without salting out (DI-HiSorb NS), Direct Immersion HiSorb with salting out (DI-HiSorb S), Headspace HiSorb without salting out (HS-HiSorb NS), Headspace HiSorb with salting out (HS-HiSorb S), Thermal Desorption without salting out (TD-NS), Thermal Desorption with salting out (TD S), Headspace Solid Phase Microextraction without salting out (HS-SPME NS), Headspace Solid Phase Microextraction with salting out (HS-SPME S).

Figure 2a,b are principal component analysis (PCA) plots, highlighting the associations of the different extraction techniques with each chemical class. The chemical class data used to generate the PCA was based on the percentage of each chemical class, rather than individual VOCs determined for each technique (with and without salting out for each GC column polarity), to visualize the associations between chemical class and individual extraction techniques, rather than individual VOC. Figure 2a relates to each chemical class with and without salting out using the non-polar GC column. The total level of discrimination was 44.8% (PCA 1 23.6% and PCA 2 20.6%). It is immediately apparent that salting out did not have a major impact on the individual extraction techniques, as with and without salting out (S and NS) for each technique are very closely associated with each other. Both DI-HiSorb S and DI-HiSorb NS were most strongly associated with lactones as previously mentioned, and were separate from HS-HiSorb S and HS-HiSorb NS, which were not strongly associated with any chemical class, but more so with esters and ketones. Although both HS-HiSorb techniques appeared closely associated with the chemical group acids, this was more a reflection of using the overall percentage of each chemical class data, the fact that only one VOC (acetic acid) made up this chemical class, and due to the relationship of this acid with the other extraction techniques. In fact, HS-HiSorb NS did not extract acetic acid. TD (TD S and TD NS) were most strongly associated with aldehydes, benzene/phenols, alcohols, and ketones, while HS-SPME (HS-SPME S and HS-SPME NS) were most closely associated with terpenes and sulphur compounds. Although the HS-SPME techniques did extract a high percentage of ketones and aldehydes, the association was less obvious as the relationship of these chemical classes to the other extraction techniques also influenced their position on the PCA (as stated earlier, the overall percentage of each chemical class data was used to generate the PCA rather than individual VOC).

Figure 2b highlights the same associations of chemical classes with each extraction technique with and without salting out, but using the polar GC column. The level of overall discrimination was less, at 39.7% (PCA 1 22.2% and PCA 2 17.5%), than that achieved in Figure 2a. As acetic acid was found using the polar GC column, the acid chemical class is not present. Some similar patterns are evident with the polar GC column as found with the non-polar GC column. It is also evident that the impact of salting out was minimal due to the close association of each individual technique with and without salting out (S and NS). For the polar GC column, both DI-HiSorb (DI-HiSorb S and DI-HiSorb NS) and HS-HiSorb (HS-HiSorb S and HS-HiSorb NS) were much more closely associated with lactones and furans. However, there were some anomalies in that DI-HiSorb, independent of salting out, had little or no recovery of furans, and that both DI-HiSorb and HS-HiSorb recovered significant levels of ketones, which were not reflected in the PCA. As previously mentioned, this is due to the use of the percentage of chemical class data rather than individual VOC data to create the PCA. HS-SPME (HS-SPME S and HS-SPME NS) were most closely associated with sulphur compounds, terpenes, and esters, but also with aldehydes and ketones. TD (TD S and TD NS) were most closely associated with alcohols and benzene/phenol VOCs, and, to a lesser extent, with aldehydes and ketones.

### 3.4. The Selectivity of Each Extraction Technique

**Figure 3:**
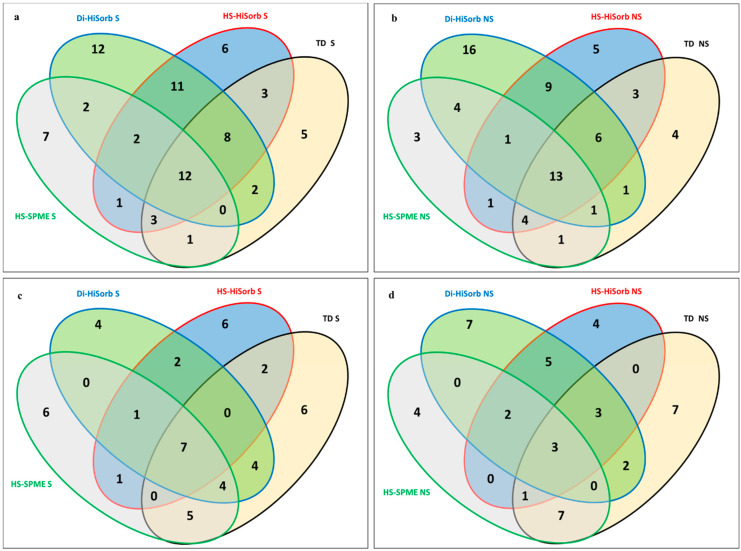
Venn diagrams of the number of volatile organic compounds extracted by each technique, including commonalities (**a**) with salting out using the non-polar GC column, (**b**) without salting out using the non-polar GC column, (**c**) with salting out using the polar GC column, and (**d**) without salting out using the polar GC column. Direct Immersion HiSorb without salting out (DI-HiSorb NS), Direct Immersion HiSorb with salting out (DI-HiSorb S), Headspace HiSorb without salting out (HS-HiSorb NS), Headspace HiSorb with salting out (HS-HiSorb S), Thermal Desorption without salting out (TD-NS), Thermal Desorption with salting out (TD S), Headspace Solid Phase Microextraction without salting out (HS-SPME NS), Headspace Solid Phase Microextraction with salting out (HS-SPME S).

Figure 3 is a series of Venn diagrams representing the selectivity of each technique in relation to (a) with salting out using the non-polar GC column, (b) without salting out using the non-polar GC column, (c) with salting out using the polar GC column, and (d) without salting out using the polar GC column. These figures highlight commonalities in relation to VOCs extracted by each technique and those recovered only by each individual extraction technique. It is immediately apparent that more VOCs were extracted using the non-polar GC columns (Figure 3a,b) than with the polar GC column (Figure 3c,d), as previously stated. However, these figures give a better insight into the discrepancies and commonalities with regard to the numbers of VOCs extracted across all four techniques. The greatest number of VOCs were associated with the two HiSorb techniques (DI-HiSorb and HS-HiSorb).

More commonalities were evident in relation to specific VOCs between both Hi-Sorb techniques, which is not surprising as they both utilize the same sorbent phase (PDMS), despite the fact that, in general, PDMS is regarded as less useful for the recovery of polar VOCs [26]. Thus overall, Hi-Sorb was quite effective for the general recovery of VOCs in WMP. Considerably fewer synergies were evident between both Hi-Sorb techniques and HS-SPME and TD. As mentioned, PDMS is thought to be more effective for the recovery of less polar VOCs than HS-SPME with multiple fiber phases [23]. HS-SPME appeared to be very effective at recovering terpenes and sulphur compounds independent of column polarity and salting out. While quite poor at recovering lactones, furan, and acids, this was also dependent upon GC column polarity and, to a much lesser extent, salting out. The DVB/CAR/PDMS multiphase SPME fibers tended to extract very volatile low boiling point VOCs more effectively [25]. In addition, as DVB is a polar porous coating, it is quite efficient in extracting polar compounds and, thus, was useful for sulphur VOCs [19,25,27], as evident in this study. The TEN components of the TD phase were less useful for very volatile VOCs, but compensated to some extent by the inclusion of CAR in the packing material [24].

In summary, 12 VOCs were extracted by every technique (1-pentanol, 1-hexanol, hexanal, heptanal, octanal, nonanal, benzaldehyde, toluene, 2-heptanone, 2-nonanone, dimethyl sulfide, and D-limonene) (Table 2a) with salting out using the non-polar GC column (Figure 3a). Thirteen 13 VOCs (1-pentanol, hexanal, heptanal, octanal, nonanal, benzaldehyde, toluene, benzene, 2-pentanone, 2-heptanone, methyl isopropyl ketone, 2-nonanone, and D-limonene) (Table 2a) were extracted by every technique without salting out using the non-polar GC column (Figure 3b). Whereas, in relation to the polar GC column, only seven VOCs (pentanal, heptanal, nonanal, benzaldehyde, decanal, acetone, and 2-heptanone) (Table 2b) were extracted with salting out (Figure 3c), and only three VOCs (heptanal, benzaldehyde, and acetone) (Table 2b) without salting out (Figure 3d).

The only VOCs extracted by all four extraction techniques independent of GC column polarity and salting out were heptanal and benzaldehyde (Table 2a,b). This likely reflects a combination of their relative abundance and chemical properties, which enabled them to be more easily recovered by each technique, despite the range of different phases and GC column polarities.

### 3.5. The Abundance of Volatile Organic Compounds in Whole Milk Powder by Each Extraction Technique

The greatest abundance of VOCs extracted across all techniques was achieved by DI-HiSorb NS, independent of GC column polarity (Table 4). As mentioned, all other abundances were expressed as a percentage of the technique with the greatest abundance (i.e., DI-HiSorb NS equated to 100% abundance). The abundance of VOCs recovered by DI-HiSorb NS was also impacted by GC column polarity, as abundances achieved by the non-polar GC column were ~41% lower than that achieved by the polar GC column (data not shown). Therefore, even though more VOCs were recovered using the non-polar GC column by DI-HiSorb NS, the total abundances were lower. The average total abundance for DI-HiSorb S, HS-HiSorb NS, HS-HiSorb S, HS-SPME NS, TD NS, HS-SPME S and TD S for the non-polar GC column was 34.2%, 11.6%, 7.5%, 3.9%, 2.2%, 1.7%, and 1.3%, respectively. A similar trend was evident for the polar GC column, where DI-HiSorb S, HS-HiSorb NS, HS-HiSorb S, TD NS, TD S, HS-SPME NS, and HS-SPME S were 39.7%, 7.7%, 4.0%, 3.1%, 2.5%, 1.3%, and 1.2%, respectively, of that attained by DI-HiSorb NS (100%). The much greater abundance of the DI-HiSorb technique appears mainly due to the advantages of DI over HS, namely, the high capacity of the phase (which is much greater than SPME), and the selectivity of the phases’ ability to extract lactones (more volume and quantities of lactones). The abundance of DI-HiSorb was impacted by salting out, as the addition of salt decreased abundances by approximately two thirds. The abundances of TD and HS-SPME were similar and slightly less than those achieved for HS-HiSorb. Therefore, the dynamic nature of TD, in comparison to the static HS-SPME and HS-HiSorb techniques, did not significantly impact VOC abundance. Differences in capacity and selectivity of the difference phases had a lesser impact than DI versus HS on abundance. A study comparing the SBSE (similar to DI-HiSorb), HSSE (similar to HS-HiSorb), and HS-SPME also found a similar trend for the extraction of fruit VOCs [28], in that SBSE extracted more and a greater abundance of VOCs than HSSE and HS-SPME.

### 3.6. The Reproducibility of Each Extraction Technique

The reproducibility of each technique was assessed by comparing the average percent relative standard deviation, (average of the percent relative standard deviation of every VOC for each technique) (Table 4) for each extraction technique in relation to column polarity and salting out. In terms of the non-polar GC column with and without salting out, the average standard deviation varied from 33.5% (HS-HiSorb S) to 45.3% (DI-HiSorb S). The average standard deviation range was greater for the polar GC column with or without salting out, from 32.5% for TD NS to 90.0% for HS-HiSorb NS. Overall reproducibility was lower for the HS techniques (HS-HiSorb S, HS-SPME NS, and HS-HiSorb NS) for the polar GC column than any of the other techniques. A recent study on spray dried sheep milk found that the average reproducibility (again, based on average relative standard deviation) of HS-SPME and SBSE was better than HSSE using a non-polar column [19].

It must be stated that the average percent relative standard deviation is a relatively crude approach to assess reproducibility. Nevertheless, it was used in this study for comparative convenience across the four techniques due to the number of factors assessed and the significant number of VOCs extracted. The average percentage relative standard deviation does not account for differences in the numbers, abundances, or the selectivity of each technique (impacted by the chemical properties of VOCs and the phases used in each extraction technique) all of which can have an impact on reproducibility. Thus, the individual relative percentage standard deviation values attained for each VOC across each technique with or without salting out for each GC column polarity provided a more in-depth, true reflection of reproducibility (Table 2a,b).

## 4. Conclusions

The evaluation of WMP by these four extraction techniques has highlighted the extent of VOCs present, which consisted mainly of ketones, aldehydes, lactones, and alcohols with lower numbers of esters, benzenes, phenols, furans, terpenes, sulphur compounds, and one acid. The overall difference in selectivity between the extraction techniques also highlights the need for multiple extraction techniques in order to obtain as true a representation of the complete volatile profile as possible. This is a simple fact, but often forgotten in volatile research of dairy and other foods. In relation to the four techniques, DI-HiSorb, HS-HiSorb, TD, and HS-SPME, the impact of GC column polarity was far greater than the impact of salting out under the conditions evaluated. It would appear that, unless specifically required to target a VOC (or specific VOCs) using a polar GC column, significantly more VOC information can be attained than using a non-polar GC column. As stated, the impact of salting out was minimal, but did vary depending upon the extraction technique, GC column polarity, and in relation to individual VOCs. Overall, the greatest number of VOCs were extracted by DI-HiSorb using the non-polar GC column, and slightly more without salting out. However, even though the numbers of VOCs extracted by DI-HiSorb was considerably reduced using the polar GC column, the overall abundance of VOCs was higher than achieved with the non-polar GC column. A key element as to why the overall abundances and numbers of VOCs were generally higher with DI-HiSorb, as opposed to the other techniques, was the ability of DI-HiSorb to extract large quantities and volumes of lactones. Only TD failed to extract any lactones in these WMP samples. As HS-HiSorb has the same sorptive PDMS phase as DI-HiSorb, the different conditions between DI and HS was a key factor influencing the effectiveness of each of these techniques in extracting lactones and other VOCs. It appears that it was more difficult to extract some VOCs using HS than DI, possibly due to their affinity with sample components adversely impacting their phase transition from a liquid to the gas phase during HS analysis, likely exacerbated by higher molecular weight VOCs. It is possible that the importance of lactones in many dairy products may have been underestimated due to the widespread use of HS-SPME DVB/CAR/PDMS and CAR/PDMS phases, where the CAR component may exclude some higher molecular weight lactones [29]. Differences in the apparent capacities of the phases associated with the different techniques did not have as much of an impact on VOC extraction as the difference between DI and HS. Differences between dynamic HS (TD) and static HS (HS-HiSorb and HS-SPME) techniques also did not significantly influence VOC extraction in terms of numbers and abundance. The reproducibility of most of the techniques, as assessed by the average relative percentage deviation, were similar, apart from HS-HiSorb, independent of salting out using the polar GC column, which was much diminished. However, reproducibility was very much VOC-dependent and also influenced by salting out and GC column polarity. Thus, in this study, differences between the techniques were impacted more by the choice of DI or HS, phase composition, and GC column polarity than phase capacity or salting out.

## Figures and Tables

**Table 1:** Details and codes used to describe each extraction technique with and without salting out evaluated.

Code	Description
HS-SPME S	Head space solid phase microextraction with salting out
HS-SPME NS	Head space solid phase microextraction without salting out
TD S	Thermal desorption with salting out
TD NS	Thermal desorption without salting out
DI-HiSorb S	Direct Immersion HiSorb with salting out
DI-HiSorb NS	Direct Immersion HiSorb without salting out
HS-HiSorb S	Head space HiSorb with salting out
HS-HiSorb NS	Head space HiSorb without salting out

**Table 2:** (**a**) Identification of volatile compounds by each extraction technique with and without salting out using the non-polar GC column. (**b**) Identification of volatile compounds by each extraction technique with and without salting out using the polar GC column.

(a)																					
			RI		Identification	DI-HISorb S		DI-HISorb NS		HS-HiSorb S		HS-HiSorb NS		TD S		TD NS		HS-SPME S		HS-SPME NS	
No	Compound	CAS	ORI	REF	Methods	% Area	% Stdev	% Area	% Stdev	% Area	% Stdev	% Area	% Stdev	% Area	% Stdev	% Area	% Stdev	% Area	% Stdev	% Area	% Stdev
		Alcohols																			
1	Ethanol	64-17-5	436	426	MS, RI, STD	0.73	0.16	0.27	0.26	0.72	0.31	0.53	0.38	nd	nd	nd	nd	nd	nd	nd	nd
2	1-Butanol	71-36-3	655	675	MS, RI, STD	nd	nd	nd	nd	1.06	0.35	nd	nd	nd	nd	nd	nd	nd	nd	nd	nd
3	1-Pentanol	71-41-0	762	768	MS, RI, STD	0.47	0.22	0.11	0.04	1.3	0.24	1.44	1.03	3.01	0.63	2.07	0.84	2.33	0.43	1.68	0.7
4	1-Hexanol	111-27-3	865	868	MS, RI, STD	0.17	0.07	nd	nd	1.03	0.46	0.72	0.025	1.17	0.37	0.46	0.23	0.87	0.33	0.77	0.3
5	2-Butoxy-ethanol	111-76-2	903	901	MS, RI	0.12	0.04	nd	nd	0.47	0.1	nd	nd	nd	nd	nd	nd	nd	nd	nd	nd
6	1-Octen-3-ol	3391-86-4	977	981	MS, RI	0.07	0.04	nd	nd	nd	nd	0.39	0.31	nd	nd	nd	nd	nd	nd	nd	nd
7	2-Ethyl-1-hexanol	104-76-7	1026	1030	MS, RI, STD	0.31	0.14	0.25	0.07	1.96	0.37	nd	nd	8.61	1.67	7.2	1.37	nd	nd	nd	nd
8	1-Octanol	111-87-5	1067	1071	MS, RI, STD	0.22	0.15	nd	nd	nd	nd	nd	nd	nd	nd	nd	nd	nd	nd	nd	nd
9	α-Terpineol	10482-56-1	1198	1192	MS, RI	nd	nd	nd	nd	nd	nd	0.57	0.28	0.32	0.07	0.26	0.04	nd	nd	nd	nd
		Aldehydes																			
10	Acrolein	107-02-8	449	470	MS, RI	0.44	0.22	1.88	0.41	nd	nd	1.26	0.61	nd	nd	nd	nd	nd	nd	nd	nd
11	Butanal	123-72-8	578	596	MS, RI, STD	0.22	0.14	0.09	0.09	nd	nd	nd	nd	nd	nd	nd	nd	5	3.23	nd	nd
12	3-Methyl butanal	590-86-3	647	654	MS, RI, STD	nd	nd	nd	nd	0.61	0.21	0.56	0.19	1.31	0.41	1.33	0.56	4.58	1.41	5.35	2.65
13	Pentanal	110-62-3	697	697	MS, RI, STD	0.9	0.84	0.34	0.11	nd	nd	nd	nd	nd	nd	nd	nd	32.05	12.67	nd	nd
14	Hexanal	66-25-1	799	801	MS, RI, STD	0.94	0.48	0.65	0.26	7.42	4.64	7.45	5.21	6.64	1.66	6.95	2.62	11.43	8.06	16.66	9.82
15	4-Heptenal,(Z)-	6728-31-0	895	902	MS, RI	nd	nd	nd	nd	0.45	0.08	nd	nd	nd	nd	nd	nd	0.32	0.13	nd	nd
16	Heptanal	111-71-7	900	901	MS, RI, STD	0.69	0.15	0.59	0.08	7.11	1.68	7.73	2.42	8.48	1.38	9.38	2.32	5.87	1.93	9.89	2.19
17	Benzaldehyde	100-52-7	967	960	MS, RI, STD	0.15	0.04	0.2	0.05	1.3	0.6	1.86	0.4	1.13	0.37	1.81	1.18	0.34	0.14	1.26	0.12
18	Octanal	124-13-0	1002	1004	MS, RI, STD	0.31	0.08	0.29	0.06	3.21	0.94	3.08	1.09	3.49	0.99	3.78	2.13	0.71	0.12	1.77	0.46
19	Benzeneacetaldehyde	122-78-1	1048	1048	MS, RI, STD	nd	nd	0.04	0.01	nd	nd	1.43	0.64	nd	nd	nd	nd	nd	nd	nd	nd
20	2-Octenal,(E )-	2548-87-0	1059	1057	MS, RI	0.07	0.01	0.1	0.04	nd	nd	nd	nd	0.22	0.09	0.3	0.2	nd	nd	nd	nd
21	Nonanal	124-19-6	1103	1106	MS, RI, STD	1.27	0.39	1.18	0.21	17.52	3.1	16.91	3.47	17.93	2.58	18.26	3.65	1.5	0.24	3.27	0.32
22	2-Nonenal,(E )-	18829-56-6	1160	1160	MS, RI	0.17	0.11	0.11	0.04	0.41	0.13	nd	nd	0.22	0.15	0.2	0.06	nd	nd	nd	nd
23	Decanal	112-31-2	1204	1205	MS, RI, STD	0.36	0.1	0.28	0.1	5.27	1.96	4.57	1.4	1.24	0.46	1.76	0.79	nd	nd	nd	nd
24	2-Decenal,(E )-	3913-81-3	1262	1266	MS, RI	0.12	0.09	0.05	0.01	1.32	0.29	nd	nd	0.11	0.09	0.22	0.21	nd	nd	nd	nd
25	Undecanal	112-44-7	1306	1309	MS, RI, STD	0.07	0.02	0.05	0.02	0.49	0.06	0.48	0.11	0.12	0.03	0.21	0.06	nd	nd	nd	nd
26	2-Undecenal	2463-77-6	1364	1350	MS, RI, STD	0.2	0.09	nd	nd	nd	nd	nd	nd	nd	nd	nd	nd	nd	nd	nd	nd
27	Dodecanal	112-54-9	1407	1401	MS, RI, STD	0.12	0.03	0.11	0.03	0.41	0.03	0.53	0.1	0.25	0.07	0.3	0.17	nd	nd	nd	nd
		Benzene/Phenols																			
28	Benzene	71-43-2	658	669	MS, RI, STD	0.36	0.17	0.31	0.2	nd	nd	1.33	0.47	6.77	3.33	8.08	5.53	nd	nd	6.2	2.7
29	Toluene	108-88-3	766	763	MS, RI, STD	0.1	0.02	0.09	0.05	0.51	0.24	0.6	0.12	4.54	0.73	3.64	1.08	1.72	0.67	1.6	1.07
30	p-Xylene	106-42-3	870	867	MS, RI, STD	nd	nd	nd	nd	nd	nd	0.77	0.78	3.39	0.6	3.14	0.8	nd	nd	nd	nd
31	o-xylene	95-47-6	898	900	MS, RI, STD	nd	nd	nd	nd	nd	nd	nd	nd	1.74	0.75	1.23	0.34	nd	nd	nd	nd
32	Benzyl alcohol	108-95-2	974	995	MS, RI, STD	nd	nd	nd	nd	0.61	0.13	nd	nd	nd	nd	0.23	0.09	nd	nd	nd	nd
33	Phenol	100-51-6	1035	1037	MS, RI, STD	0.16	0.1	0.12	0.04	0.79	0.23	0.76	0.17	0.73	0.31	0.66	0.12	nd	nd	nd	nd
		Ketones																			
34	Acetone	67-64-1	451	496	MS, RI, STD	1.62	0.61	nd	nd	8.11	2.62	6.5	1.02	nd	nd	nd	nd	5.4	0.65	nd	nd
35	Diacetyl	431-03-8	548	596	MS, RI, STD	nd	nd	nd	nd	nd	nd	1.75	0.57	3.72	1.67	3.1	0.68	nd	nd	nd	nd
36	Hydroxyacetone	116-09-6	663	657	MS, RI	0.68	0.31	1.03	0.71	0.12	0.11	2.91	2.49	nd	nd	nd	nd	nd	nd	nd	nd
37	2-Pentanone	107-87-9	684	687	MS, RI, STD	nd	nd	0.26	0.08	2.19	0.3	5.38	3.13	4.1	0.53	3.46	0.59	1.21	1.16	5.52	0.91
38	2-Butanone	108-10-1	733	740	MS, RI, STD	nd	nd	nd	nd	1.82	0.39	1.25	0.53	10.04	1.65	7.3	0.83	nd	nd	4.41	1.19
39	Methyl Isobutyl Ketone	108-10-1	735	740	MS, RI, STD	nd	nd	0.09	0.02	0.58	0.11	0.5	0.23	1.48	0.8	0.93	0.23	0.67	0.13	0.76	0.46
40	2-Hexanone	591-78-6	789	790	MS, RI, STD	nd	nd	nd	nd	nd	nd	nd	nd	nd	nd	nd	nd	0.67	0.04	nd	nd
41	2-Heptanone	110-43-0	887	891	MS, RI, STD	1.23	0.28	1.14	0.27	11.95	0.97	10.34	3.18	2.62	0.41	3.85	0.6	13.12	2.71	16.17	6.16
42	2,3-Octanedione	585-25-1	981	967	MS, RI, STD	0.17	0.05	0.16	0.04	1.56	0.37	nd	nd	nd	nd	nd	nd	nd	nd	nd	nd
43	3-Octanone	106-68-3	982	989	MS, RI	0.05	0.02	0.04	0.02	nd	nd	nd	nd	nd	nd	nd	nd	nd	nd	nd	nd
44	2-Octanone	111-13-7	988	992	MS, RI, STD	nd	nd	0.07	0.02	0.39	0.16	0.45	0.07	0.56	0.13	0.55	0.28	nd	nd	nd	nd
45	3,5-Octadien-2-one,(E,E)-	30086-02-3	1069	1072	MS, RI	0.46	0.06	0.37	0.06	1.44	0.38	1.33	0.22	nd	nd	nd	nd	nd	nd	nd	nd
46	Acetophenone	98-86-2	1070	1079	MS, RI, STD	0.09	0.02	nd	nd	0.65	0.13	0.52	0.07	0.68	0.25	nd	nd	nd	nd	nd	nd
47	3,5-Octadien-2-one	38284-27-4	1076	1072	MS, RI	nd	nd	nd	nd	nd	nd	nd	nd	nd	nd	0.69	0.48	0.92	0.23	0.45	0.13
48	2-Nonanone	821-55-6	1088	1094	MS, RI, STD	0.3	0.09	0.51	0.12	3.19	0.19	3.43	0.18	0.54	0.12	0.69	0.05	0.93	0.1	2.33	0.21
49	2-Undecanone	112-12-9	1295	1294	MS, RI, STD	nd	nd	0.08	0.02	nd	nd	0.77	0.08	nd	nd	0.15	0.02	1.64	0.26	nd	nd
50	2-Tridecanone	593-08-8	1494	1480	MS, RI, STD	nd	nd	0.27	0.14	nd	nd	nd	nd	nd	nd	nd	nd	nd	nd	nd	nd
51	2-Pentadecanone	2345-28-0	1695	1689	MS, RI, STD	0.41	0.4	0.4	0.26	nd	nd	nd	nd	nd	nd	nd	nd	nd	nd	nd	nd
52	2-Heptadecanone	2922-51-2	1897	1878	MS, RI, STD	nd	nd	0.54	0.09	0.98	0.39	nd	nd	nd	nd	nd	nd	nd	nd	nd	nd
		Lactones																			
53	γ-Crotonolactone	497-23-4	912	916	MS, RI	0.21	0.11	0.19	0.11	0.89	0.3	0.92	0.21	nd	nd	nd	nd	nd	nd	nd	nd
54	δ-Caprolactone	823-22-3	1097	1084	MS, RI	nd	nd	0.1	0.03	nd	nd	nd	nd	nd	nd	nd	nd	nd	nd	nd	nd
55	δ-Octalactone	698-76-0	1288	1288	MS, RI	nd	nd	0.63	0.4	nd	nd	nd	nd	nd	nd	nd	nd	nd	nd	nd	nd
56	δ-Nonalactone	3301-94-8	1394	1404	MS, RI	0.22	0.1	0.14	0.03	nd	nd	nd	nd	nd	nd	nd	nd	nd	nd	nd	nd
57	δ-Decalactone	705-86-2	1502	1506	MS, RI	31.87	2.9	28.34	4.87	3.81	0.5	3.06	0.45	nd	nd	nd	nd	0.29	0.05	1.05	0.22
58	δ-undecalactone	710-04-3	1602	1627	MS, RI	0.08	0.02	1.68	0.63	nd	nd	nd	nd	nd	nd	nd	nd	nd	nd	nd	nd
59	(Z) Dairy lactone	18679-18-0	1664	1675	MS, RI	0.64	0.23	nd	nd	nd	nd	nd	nd	nd	nd	nd	nd	nd	nd	nd	nd
60	γ-Dodecalactone	2305-(05)-7	1685	1674	MS, RI	8.56	10.18	13.9	15.58	nd	nd	nd	nd	nd	nd	nd	nd	nd	nd	nd	nd
61	δ-Dodecalactone	713-95-1	1717	1719	MS, RI	29.02	3.99	40.05	7.86	1.52	0.25	1.37	0.21	nd	nd	nd	nd	nd	nd	nd	nd
62	δ-Tridecalactone	7370-92-5	1824	1778	MS, RI	0.55	0.18	1.21	0.37	nd	nd	nd	nd	nd	nd	nd	nd	nd	nd	nd	nd
63	δ-Tetradecalactone	2721-22-4	1930	1938	MS, RI	13.46	1.16	nd	nd	nd	nd	nd	nd	nd	nd	nd	nd	nd	nd	nd	nd
		Sulfurous Compounds																			
64	Dimethyl sulfide	75-18-3	519	510	MS, RI, STD	nd	nd	nd	nd	nd	nd	nd	nd	nd	nd	nd	nd	1.68	0.65	3.93	1.68
65	Dimethyl disulfide	624-92-0	743	739	MS, RI, STD	0.09	0.02	0.1	0.02	0.19	0.09	nd	nd	1.74	0.32	4.62	1	1.7	0.86	0.85	0.15
		Terpenes																			
66	α-Pinene	80-56-8	939	930	MS, RI, STD	nd	nd	nd	nd	nd	nd	nd	nd	nd	nd	nd	nd	0.58	0.47	1.93	1.24
67	3-Carene	13466-78-9	1015	1009	MS, RI, STD	nd	nd	nd	nd	nd	nd	nd	nd	nd	nd	nd	nd	0.49	0.17	1.85	1.02
68	D-Limonene	5989-27-5	1032	1022	MS, RI, STD	0.01	0.02	0.05	0.01	0.33	0.06	0.28	0.06	2.52	2.55	1.18	0.89	0.27	0.04	0.3	0.13
69	Longifolene	475-20-7	1439	1432	MS, RI, STD	nd	nd	nd	nd	nd	nd	nd	nd	0.14	0.03	0.17	0.08	nd	nd	nd	nd
		Acids																			
70	Acetic acid	64-19-7	535	629	MS, RI, STD	0.21	0.05	0.23	0.14	1.17	1.3	nd	nd	nd	nd	nd	nd	nd	nd	nd	nd
		Esters																			
71	Methyl butanoate	623-42-7	716	724	MS, RI, STD	nd	nd	nd	nd	nd	nd	1.05	0.3	0.22	0.04	1.16	0.4	1.5	0.55	2.44	1.14
72	Methyl pyruvate	108-10-1	735	740	MS, RI	0.29	0.19	0.16	0.08	0.54	0.36	nd	nd	nd	nd	nd	nd	nd	nd	nd	nd
73	Ethylbenzene	100-41-4	859	851	MS, RI, STD	nd	nd	nd	nd	0.53	0.11	nd	nd	0.22	0.15	0.68	0.41	nd	nd	nd	nd
74	Methy hexanoate	106-70-7	918	922	MS, RI	nd	nd	nd	nd	nd	nd	1.31	0.41	nd	nd	nd	nd	0.83	0.1	3.36	1.88
75	Methyl octanoate	111-11-5	1117	1126	MS, RI	nd	nd	nd	nd	nd	nd	0.53	0.43	nd	nd	nd	nd	nd	nd	nd	nd
76	Methyl hexadecanoate	112-39-0	1915	1909	MS, RI	nd	nd	nd	nd	2.16	0.77	2	0.32	nd	nd	nd	nd	nd	nd	nd	nd
		Furans																			
77	2-Methyl-furan	534-22-5	602	604	MS, RI, STD	nd	nd	0.17	0.04	0.52	0.42	nd	nd	nd	nd	nd	nd	nd	nd	7.59	4.83
78	2-Pentyl-furan	3777-69-3	989	991	MS, RI, STD	nd	nd	0.05	0.01	0.43	0.19	0.44	0.08	nd	nd	nd	nd	nd	nd	nd	nd
79	2-Furanmethanol	98-0-0	851	850	MS, RI, STD	0.65	0.59	0.59	0.75	0.49	0.35	nd	nd	nd	nd	nd	nd	nd	nd	nd	nd
80	Furfural	98-01-1	833	852	MS, RI, STD	0.39	0.19	0.36	0.35	0.51	0.11	0.93	0.24	nd	nd	nd	nd	nd	nd	nd	nd
**(b)**																					
		Alcohols																			
1	α-Terpineol	10482-56-1	1206	1192	MS, RI	nd	nd	nd	nd	nd	nd	nd	nd	0.73	0.29	nd	nd	nd	nd	nd	nd
2	1-Pentanol	71-41-0	1262	1250	MS, RI, STD	nd	nd	nd	nd	2.34	1.86	2.17	3.35	nd	nd	nd	nd	nd	nd	nd	nd
3	1-Hexanol	111-27-3	1365	1355	MS, RI, STD	nd	nd	nd	nd	nd	nd	nd	nd	1.11	0.4	0.88	0.38	1.44	0.86	0.87	0.91
4	1-Penten-3-ol	111-27-3	1365	1355	MS, RI	nd	nd	nd	nd	nd	nd	nd	nd	1.89	1.02	1.25	0.95	2.06	1.76	nd	nd
5	2-Butoxy-ethanol	111-76-2	1423	1405	MS, RI	nd	nd	nd	nd	nd	nd	nd	nd	nd	nd	1.45	1.4	nd	nd	nd	nd
6	1-Octen-3-ol	3391-86-4	1460	1450	MS, RI	nd	nd	nd	nd	nd	nd	nd	nd	nd	nd	0.67	0.1	nd	nd	nd	nd
7	2-Ethyl-1-hexanol	71-41-0	1502	1491	MS, RI, STD	nd	nd	nd	nd	nd	nd	nd	nd	12.02	1.89	15.69	3.3	4.18	0.51	2.18	0.81
8	1-Nonanol	143-08-8	1673	1660	MS, RI, STD	nd	nd	nd	nd	nd	nd	nd	nd	nd	nd	1.81	0.33	nd	nd	nd	nd
9	Acrolein	107-02-8	449	470	MS, RI	0.2	0.24	0.31	0.05	4.55	2.02	nd	nd	nd	nd	nd	nd	nd	nd	nd	nd
10	Butanal	123-72-8	578	596	MS, RI, STD	nd	nd	0.12	0.03	nd	nd	nd	nd	1.04	0.08	1.3	0.24	nd	nd	nd	nd
11	3-Methyl-butanal	590-86-3	652	654	MS, RI, STD	0.28	0.05	nd	nd	nd	nd	nd	nd	0.59	0.48	1.08	0.65	2.2	1.13	17.4	17.85
12	Pentanal	110-62-3	994	979	MS, RI, STD	0.68	0.26	0.52	0.23	1.68	1.05	2.22	1.93	7.38	2.74	nd	nd	11.23	5.97	nd	nd
13	Hexanal	66-25-1	1098	1083	MS, RI, STD	nd	nd	nd	nd	nd	nd	nd	nd	2.6	0.64	3.87	0.58	5.9	0.33	4.55	6.02
14	Heptanal	111-71-7	1204	1184	MS, RI, STD	0.57	0.1	0.4	0.13	9.62	4.17	13.91	6.8	4.01	0.92	4.81	0.89	10.47	3.64	24.2	10.48
15	Octanal	124-13-0	1310	1289	MS, RI, STD	nd	nd	0.4	0.06	2.34	0.43	4.32	0.98	1.85	0.78	2.43	1.09	nd	nd	nd	nd
16	Nonanal	124-19-6	1416	1391	MS, RI, STD	1.02	0.24	0.89	0.31	1.27	0.96	8.41	6.43	10.12	3.05	12.09	2.97	0.62	0.37	nd	nd
17	Decanal	112-31-2	1523	1498	MS, RI, STD	0.48	0.12	0.61	0.31	16.88	9.22	8.54	12.98	1.44	0.24	2.99	1.03	nd	nd	nd	nd
18	Benzaldehyde	100-52-7	1570	1520	MS, RI, STD	0.52	0.1	0.33	0.07	5.63	4.25	4.56	4.43	1.21	0.31	2.55	0.45	0.9	0.11	2.28	1.06
19	Dodecanal	112-54-9	1733	1710	MS, RI, STD	nd	nd	nd	nd	0.31	0.47	6.08	1.32	nd	nd	nd	nd	nd	nd	nd	nd
		Benzene/Phenols																			
20	Toluene	108-88-3	766	763	MS, RI, STD	1.1	0.17	nd	nd	nd	nd	nd	nd	0.56	0.16	0.75	0.2	0.53	0.41	2.62	0.96
21	Benzene	71-43-2	955	957	MS, RI, STD	0.34	0.2	0.26	0.17	nd	nd	nd	nd	1.49	0.37	2	0.72	nd	nd	nd	nd
22	p-Xylene	106-42-3	1155	1138	MS, RI, STD	nd	nd	nd	nd	nd	nd	nd	nd	nd	nd	0.86	0.17	nd	nd	nd	nd
23	o-Xylene	95-47-6	1156	1186	MS, RI, STD	0.35	0.04	nd	nd	nd	nd	nd	nd	2.02	1.21	nd	nd	nd	nd	nd	nd
24	Phenol	108-95-2	2042	2039	MS, RI, STD	nd	nd	nd	nd	1.14	1.37	nd	nd	nd	nd	nd	nd	nd	nd	nd	nd
		Ketones																			
25	Hydroxyacetone	116-09-6	663	657	MS, RI	2.68	1.68	2.49	1.14	nd	nd	0.51	0.79	nd	nd	nd	nd	nd	nd	nd	nd
26	2-Pentanone	107-87-9	684	687	MS, RI, STD	0.74	0.43	nd	nd	nd	nd	nd	nd	5.18	3.58	nd	nd	nd	nd	8.65	2.87
27	Methyl isopropyl ketone	108-10-1	735	740	MS, RI, STD	nd	nd	0.45	0.18	nd	nd	nd	nd	nd	nd	nd	nd	8.97	1.96	nd	nd
28	Acetone	67-64-1	825	819	MS, RI, STD	1.6	0.62	1.17	0.33	2.89	1.09	3.46	1.67	26.04	7.92	29.76	8.27	9.61	2.67	6.07	5.05
29	2-Heptanone	110-43-0	887	891	MS, RI, STD	3.04	1.15	0.36	0.1	1.44	0.51	1.84	0.79	6.33	1.89	nd	nd	17.94	4.91	nd	nd
30	2-Butanone	108-10-1	913	907	MS, RI, STD	0.28	0.17	nd	nd	nd	nd	nd	nd	6.88	2.11	8.38	2.44	5.97	4.05	5.39	1.2
31	Acetophenone	98-86-2	1070	1079	MS, RI, STD	0.43	0.07	nd	nd	nd	nd	nd	nd	0.7	0.22	nd	nd	nd	nd	nd	nd
32	2,3-Pentanedione	600-14-6	1073	1058	MS, RI	nd	nd	nd	nd	nd	nd	nd	nd	0.82	0.34	0.95	0.33	nd	nd	nd	nd
33	2-Nonanone	821-55-6	1410	1390	MS, RI, STD	1.13	0.56	0.98	0.23	nd	nd	nd	nd	0.95	0.11	1.8	0.26	2.53	0.62	3.63	1.59
34	3,5-Octadien-2-one	38284-27-4	1549	1522	MS, RI	nd	nd	nd	nd	11.26	11.15	7.02	5.4	0.95	0.67	0.83	0.28	nd	nd	2.29	0.94
35	3,5-Octadien-2-one,(E,E)-	30086-02-3	1551	1570	MS, RI	1.38	0.83	1	0.47	2.84	2.36	0.66	1.32	nd	nd	nd	nd	0.92	0.74	nd	nd
36	2-Undecanone	112-12-9	1622	1598	MS, RI, STD	nd	nd	0.68	0.17	nd	nd	nd	nd	nd	nd	nd	nd	nd	nd	nd	nd
		Lactones																			
37	δ-Caprolactone	823-22-3	1864	1791	MS, RI	0.25	0.24	0.33	0.09	nd	nd	nd	nd	nd	nd	nd	nd	nd	nd	nd	nd
38	δ-Octalactone	698-76-0	2037	1976	MS, RI	nd	nd	nd	nd	1.53	2.29	nd	nd	nd	nd	nd	nd	4.21	0.26	nd	nd
39	δ-Decalactone	705-86-2	2242	2190	MS, RI	nd	nd	nd	nd	12.46	3.67	14.39	3.58	nd	nd	nd	nd	nd	nd	nd	nd
40	δ-Dodecalactone	713-95-1	2570	2436	MS, RI	62.94	1.99	62.33	1.64	5.56	4.5	nd	nd	nd	nd	nd	nd	nd	nd	nd	nd
41	δ-Tetradecalactone	7370-92-5	2892	2688	MS, RI	19.61	1.57	24.59	2.66	nd	nd	nd	nd	nd	nd	nd	nd	nd	nd	nd	nd
		Sulfurous Compounds																			
42	Dimethyl disulfide	624-92-0	743	739	MS, RI, STD	0.38	0.04	nd	nd	nd	nd	nd	nd	nd	nd	nd	nd	nd	nd	nd	nd
43	Dimethyl sulfide	75-18-3	755	754	MS, RI, STD	nd	nd	nd	nd	nd	nd	nd	nd	0.36	0.45	0.69	0.34	4.24	2	3.72	0.91
		Terpenes																			
44	α-Pinene	80-56-8	1035	1028	MS, RI, STD	nd	nd	nd	nd	nd	nd	nd	nd	nd	nd	nd	nd	0.9	0.22	2.09	1.66
45	3-Carene	13466-78-9	1152	1147	MS, RI, STD	nd	nd	nd	nd	nd	nd	nd	nd	nd	nd	nd	nd	0.64	0.19	1.45	1.94
46	D-Limonene	5989-27-5	1218	1200	MS, RI, STD	nd	nd	0.16	0.07	nd	nd	nd	nd	0.8	0.41	1.1	0.27	nd	nd	7.72	4.94
		Esters																			
47	Methyl butanoate	623-42-7	1001	982	MS, RI, STD	nd	nd	nd	nd	nd	nd	nd	nd	nd	nd	nd	nd	2.46	0.87	4.9	2.72
48	Butyl acetate	123-86-4	1087	1074	MS, RI, STD	nd	nd	nd	nd	nd	nd	nd	nd	0.93	0.43	nd	nd	nd	nd	nd	nd
49	Ethylbenzene	100-41-4	1120	1129	MS, RI, STD	0.06	0.01	nd	nd	nd	nd	nd	nd	0.66	0.06	0.8	0.11	nd	nd	nd	nd
50	Methyl hexadecanoate	112-39-0	1915	1909	MS, RI	nd	nd	nd	nd	3.67	1.28	1.13	1.92	nd	nd	nd	nd	nd	nd	nd	nd
51	Methyl octanoate	111-11-5	1407	1385	MS, RI, STD	nd	nd	nd	nd	nd	nd	nd	nd	nd	nd	nd	nd	1.05	0.07	nd	nd
		Furans																			
52	2-Ethyl-furan	3208-16-0	968	950	MS, RI, STD	nd	nd	nd	nd	nd	nd	nd	nd	nd	nd	nd	nd	1.01	0.41	nd	nd
53	Furfural	98-01-1	1497	1461	MS, RI, STD	nd	nd	0.8	0.36	8.91	7.71	17.33	10.71	nd	nd	nd	nd	nd	nd	nd	nd
54	2-Furanmethanol	98-00-0	1681	1660	MS, RI, STD	nd	nd	0.81	0.73	nd	nd	nd	nd	nd	nd	nd	nd	nd	nd	nd	nd

Results are expressed as relative abundance normalized to internal standard (% area, % relative standard deviation). CAS (Chemical Abstracts Service number). MS (identity confirmed by mass spectra to an in-house library). RI (linear retention index as determined). ORI (linear retention index as determined in this study). REF (relevant linear retention index as published reference, if available). STD (an internal standard was used to confirm identification). nd (not determined).

**Table 3:** The numbers of volatile organic compounds extracted in whole milk powder samples.

No of VOCs Extracted	Non-Polar GC Column	Polar GC Column
With salting out	75	48
Without salting out	72	45
Total	80	54
Overall Total	85	

**Table 4:** Numbers, abundance, and reproducibility of volatile organic compounds in whole milk powder samples extracted by each technique with and without salting out and for polar and non-polar GC columns.

NON-POLAR GC COLUMN								
Extraction Technique	HS-SPME S	TD S	Di-HiSorb S	HS-HiSorb S	HS-SPME NS	TD NS	Di-HiSorb NS	HS-HiSorb NS
No of VOCs	28	34	49	46	25	36	51	42
Abundance %	1.7	1.3	34.2	7.5	3.9	2.2	100	11.6
Average RSD %	39.3	33.7	45.3	33.5	38.3	38.1	40.5	35.2
**POLAR GC COLUMN**								
**Extraction Technique**	**HS-SPME S**	**TD S**	**Di-HiSorb S**	**HS-HiSorb S**	**HS-SPME NS**	**TD NS**	**Di-HiSorb NS**	**HS-HiSorb NS**
No of VOCs	23	28	23	19	17	25	22	16
Abundance %	1.2	2.5	39.7	4	1.3	3.1	100	7.7
Average RSD %	37.4	39.1	37.6	71.1	63.8	32.5	34.6	90

No. of VOCs (number of volatile organic compounds). Abundance %, (the greatest abundance achieved by a single extraction technique equated to 100% and the remaining extraction techniques were expressed as a percentage thereof). Average RSD % (the average percentage relative standard deviation of all VOCs for extraction technique).

## Data Availability

Not applicable.
